# Data to support small area health impact modelling of air pollution in the United Kingdom

**DOI:** 10.1016/j.dib.2020.105148

**Published:** 2020-01-15

**Authors:** Andrew Ibbetson, Phil Symonds, Emma Hutchinson

**Affiliations:** a London School of Hygiene and Tropical Medicine, London, UK; b Institute of Environmental Design and Engineering, UCL, London, UK

**Keywords:** Microsimulation, Health impact modelling, Environmental risks, Deprivation, Air pollution

## Abstract

The data presented in this article were used to estimate the impacts of air pollution policies on population health and health inequalities within a spatial microsimulation model, MicroEnv [1]. They provide a basis for comparison with similar models and allow researchers to integrate additional model components without duplication of effort. Relative risk estimates for the association between air pollution and rates of ischaemic heart disease (IHD) incidence, IHD case fatality and all-cause mortality were taken from a review of the epidemiological literature and meta-analyses [2]. Modelled small area air pollution data (PM_2.5_) for Greater London, UK were obtained from an environmental consultancy. All other data were collected from open source Governmental or Non-Government Organisation (NGO) data repositories. These include all-cause mortality rates; IHD incidence, prevalence and mortality rates; general fertility rates; small area socio-economic deprivation data; and relative risk estimates for the association between deprivation and all-cause mortality.

**Table:** Specifications Table

Subject	Public Health and Health Policy.
Specific subject area	Health impact modelling of environmental risk factors at small area level.
Type of data	Graphs, Figures and Tables.
How data were acquired	All data were acquired from open access internet databases, except the modelled air pollution data and the relative risk estimates for the association between air pollution and rates of IHD incidence, IHD case fatality and all-cause mortality. The former were provided by Ricardo Energy and Environment and the latter were obtained from a review of the academic literature and meta-analyses.
Data format	Raw and Analyzed.
Parameters for data collection	All data were obtained at the highest possible spatial resolution, Lower Layer Super Output Area (LSOA), where possible. Population and health data were collected by gender and at the highest possible age resolution (single year of age if available, 5-year age groupings otherwise).
Description of data collection	Data were collected by downloading the relevant file from an open access internet database or by extraction from a review of epidemiological literature conducted by the authors.
Data source location	Greater London, United Kingdom.
Data accessibility	Repository name: MicroEnv Data identification number: 10.17632/dxrp4b656h.1 Direct URL to data: https://doi.org/10.17632/dxrp4b656h.1
Related research article	Phil Symonds, Emma Hutchinson, Andrew Ibbetson, Jonathon Taylor, James Milner, Zaid Chalabi, Michael Davies, Paul Wilkinson. MicroEnv: A microsimulation model for quantifying the impacts of environmental policies on population health and health inequalities. Science of The Total Environment https://doi.org/10.1016/j.scitotenv.2019.134105

**Table:** 

**Value of the Data** • These data allow the estimation of impacts of air pollution and other environmental policies on population health and health inequalities. • Policy makers and researchers may benefit from the insights that analysis of these data can provide, within the constraints of uncertainty. • These data provide a basis for comparison with similar models and allow researchers to integrate additional model components without duplication of effort.

## 1 Data

The data described in this article form the basis of a spatial microsimulation model, MicroEnv, which aims to investigate the impact of environmental policies on population health [1]. All data are Greater London, UK specific and are outlined in Table 1.

**Table 1:** Data sources used in the microsimulation model.

Data type	Year	Additional info	Reference
Mortality (all-cause)	2016	Period projections by year of age and gender	[3]
IHD mortality, incidence and prevalence	2016	By gender and 5-year age bands (UK)	[4]
Population	2015	Population by single year of age, gender and LSOA^a^	[5]
Socio-economic deprivation	2015	Decile of the Index of Multiple Deprivation (IMD) for each LSOA	[6]
Air pollution	2014	Annual averages of PM_2.5_ at 1 × 1 grid (mapped to LSOA)	[7]
General fertility rates	2015	Number of live births per 1000 females aged 15–44 at local authority level. Applied to the LSOA-specific female population each year	[8]

a Lower Layer Super Output Area.

### 1.1 Health data

#### 1.1.1 All-cause mortality rates

**Fig. 1:**
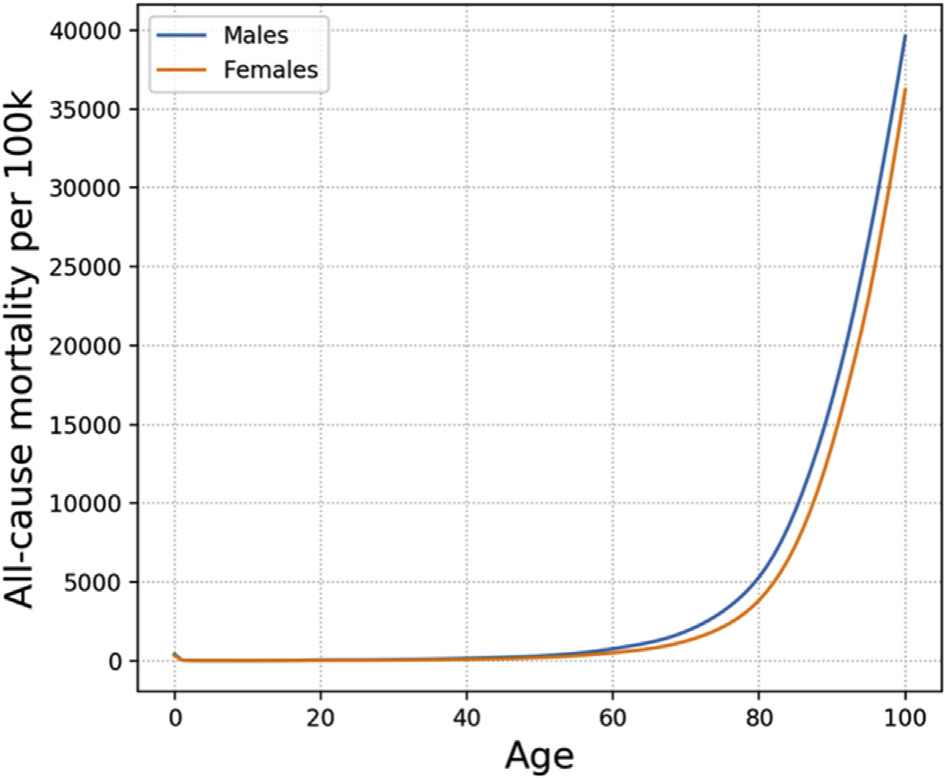
UK all-cause mortality rates (per 100k) used in MicroEnv by single year of age and sex. Source: ONS.

All-cause mortality data were obtained from the Office for National Statistics (ONS) [3]. The ONS provides both period and cohort projections by single year of age and sex for the UK. Although the model is able to take these projections into account, fixed (2016) values for mortality rates were used within MicroEnv. Fig. 1 shows all-cause mortality rates used in the model by age and sex for 2016.

#### 1.1.2 Ischaemic heart disease (IHD) incidence, prevalence and mortality rates

**Fig. 2:**
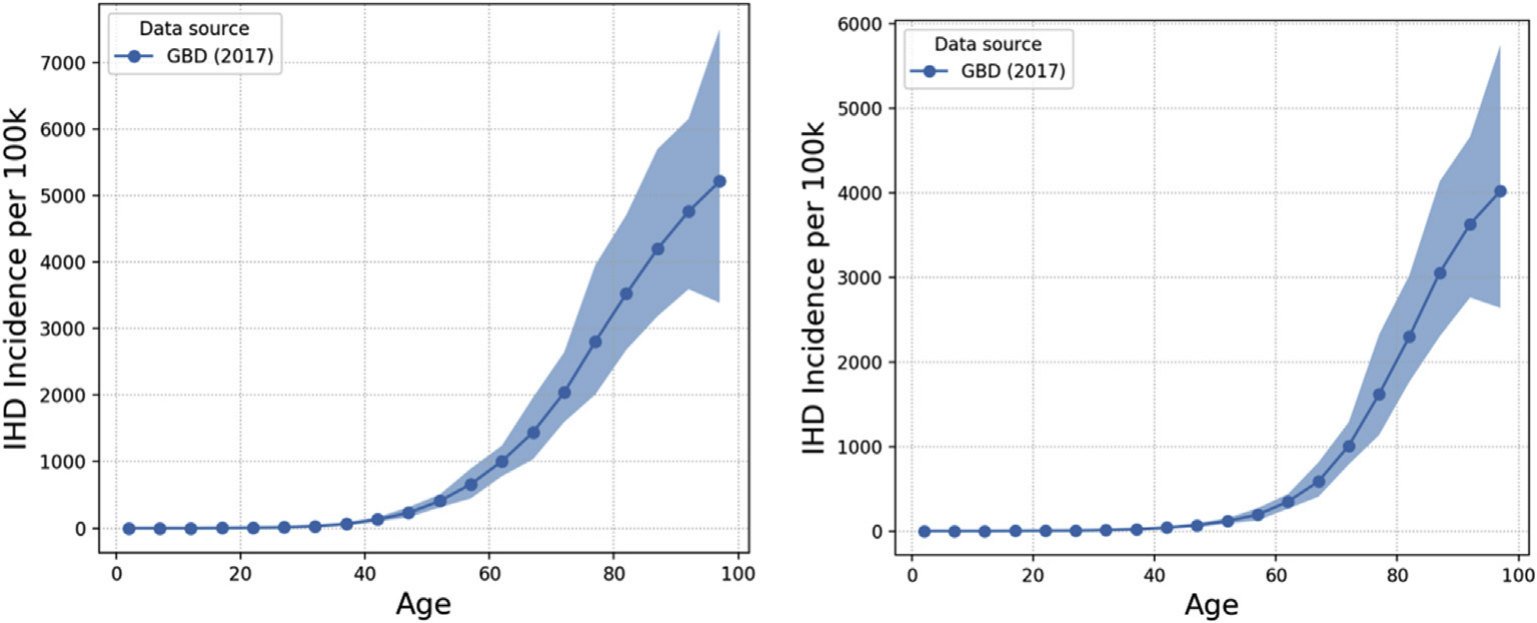
IHD incidence rates (per 100k) in England (2017) by age as output by the GBD Results Tool for males (left) and females (right). Error bands represent 95% confidence interval reported by GBD Results Tool.

**Fig. 3:**
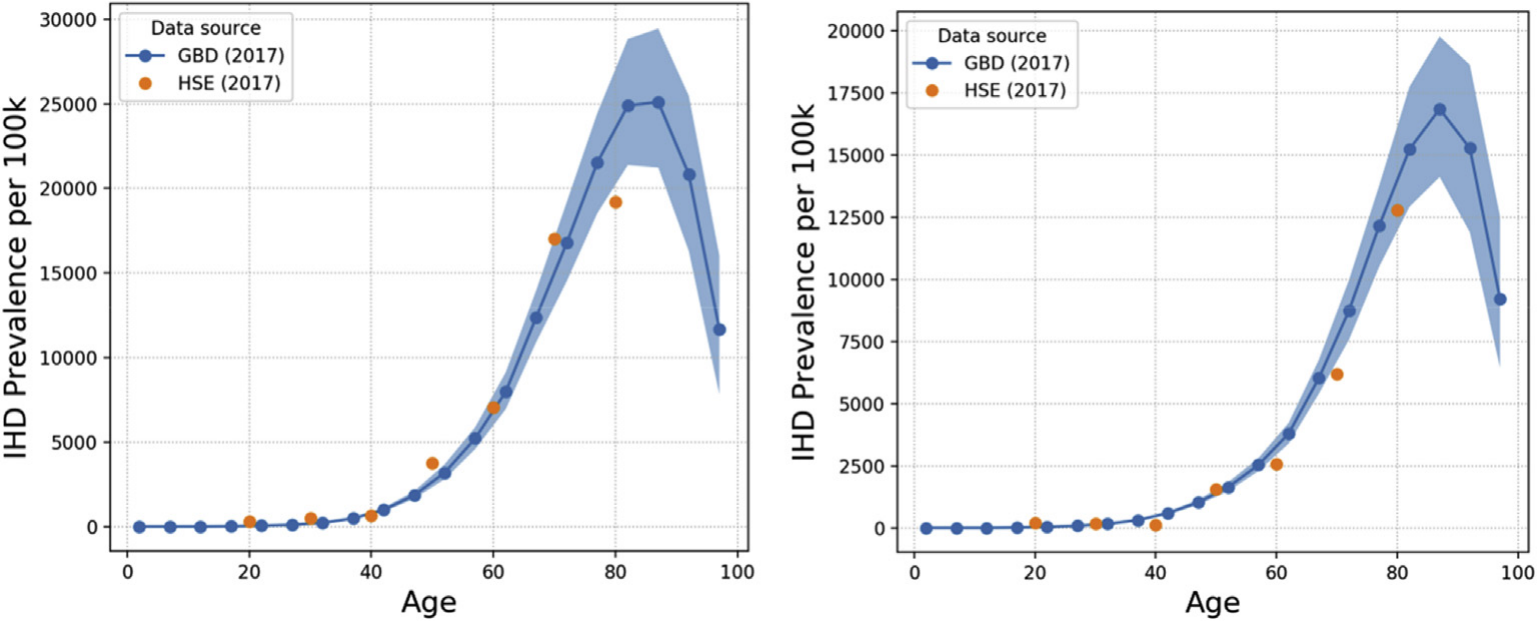
IHD prevalence rates (per 100k) in England (2017) by age as output by the GBD Results Tool compared to HSE data for males (left) and females (right). Error bands represent 95% confidence interval reported by GBD Results Tool.

**Fig. 4:**
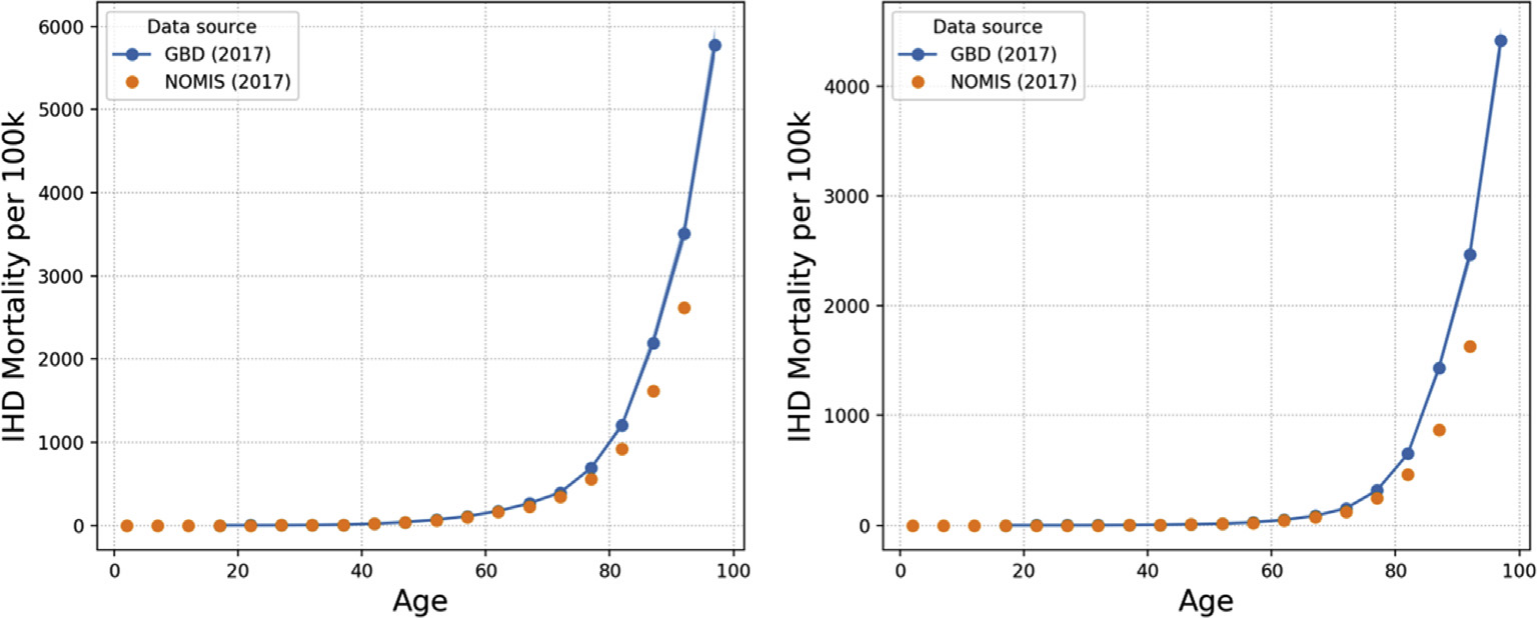
IHD mortality rates (per 100k) in England (2017) by age as output by the GBD Results Tool compared to nomis data from the ONS for males (left) and females (right).

IHD incidence, prevalence and mortality data used within MicroEnv are obtained from the GBD Results Tool [4]. Data by 5-year age band and sex for the UK (2016) were used within our model. Whilst 2016 GBD data is used in the model, 2017 data is shown here for comparison to HSE data from 2017. GBD outputs are shown with the latest Health Survey for England (HSE) data [9] overlaid, the graphs in this paper show data for England (2017). Fig. 2, Fig. 3, Fig. 4 show incidence, prevalence and mortality data, respectively. For the incidence data, it is not possible to overlay HSE data, since HSE reports incidence in terms of hospital inpatient admissions rather than new cases of IHD.

#### 1.1.3 Relative risks

Table 2 shows the relative risks used in the MicroEnv model and their source. These estimates modify the annual transition probabilities between disease states to account for the effects of air pollution and, separately, deprivation on health.

**Table 2:** Relative risks used in the microsimulation model.

Relative risk	Coefficient used	Reference
IHD incidence	1.08 per 10 μg/m^3^ (PM_2.5_)	[2]
IHD case fatality	1.21 per 10 μg/m^3^ (PM_2.5_)	[2]
All-cause mortality	1.06 per 10 μg/m^3^ (PM_2.5_)	[10]
	1.7 between most and least deprived decile (males)	[11]
	1.5 between most and least deprived decile (females)	[11]

### 1.2 Population data

**Fig. 5:**
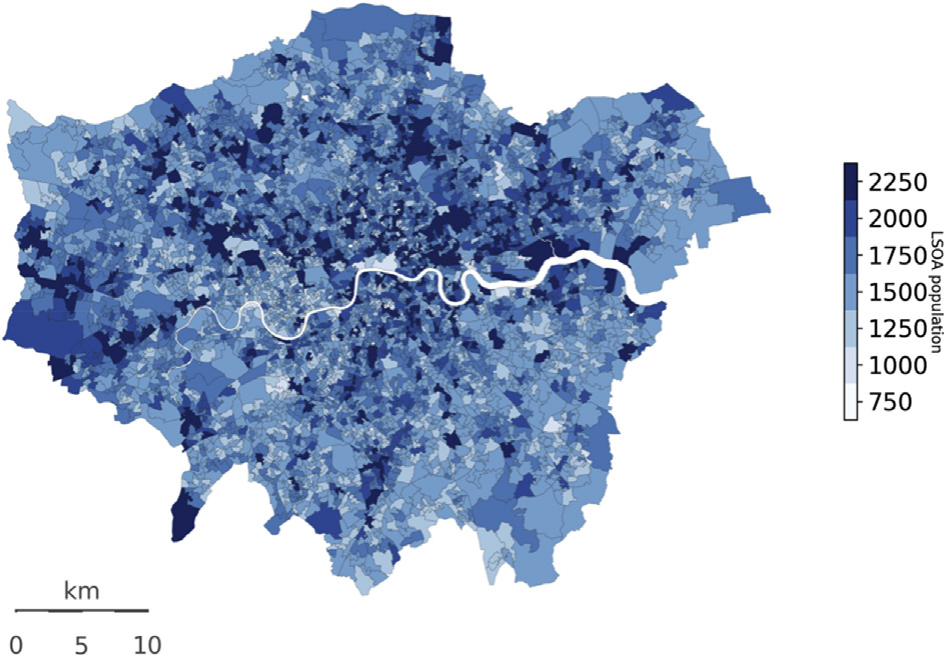
Map of population (2015) by LSOA for Greater London. Source: ONS (?).

The population data used within MicroEnv to generate an estimate of the population of Greater London were from the ONS [5]. Data is available for the 2015 population by single year of age and by sex for each LSOA in Greater London. Fig. 5 maps the estimated population of each LSOA modelled.

### 1.3 Socio-economic deprivation

**Fig. 6:**
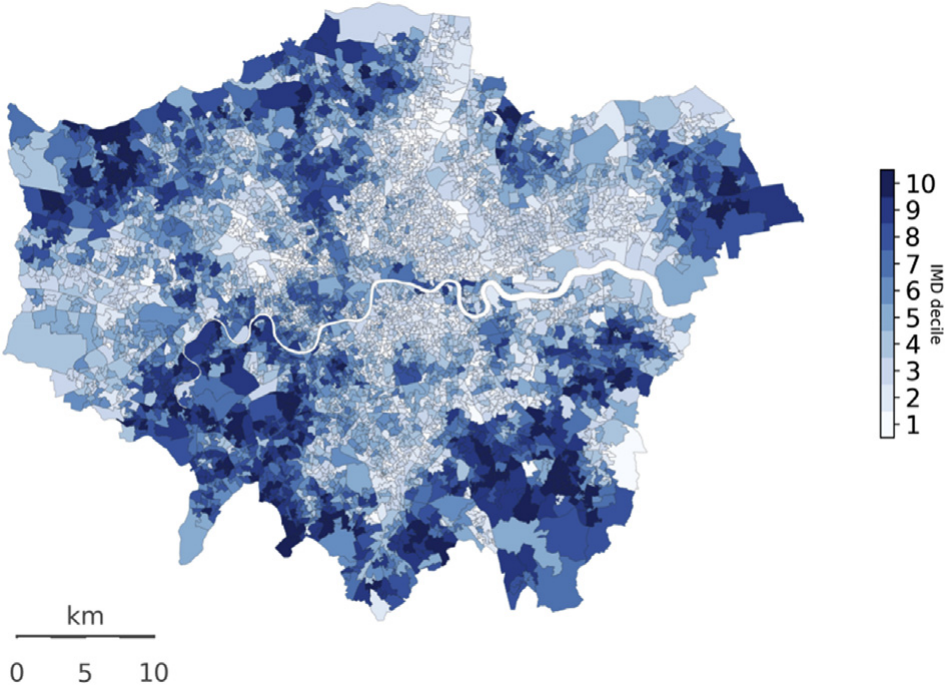
Map of IMD (2015) by LSOA for Greater London. A lower deprivation decile represents a more deprived area. Source: DCLG.

The socio-economic deprivation of individuals within each LSOA is defined using the 2015 decile of the Index of Multiple Deprivation (IMD). These are reported by the UK Department for Communities and Local Government (DCLG) [6]. Fig. 6 provides a map of IMD by LSOA for Greater London.

### 1.4 General fertility rates (GFRs)

**Fig. 7:**
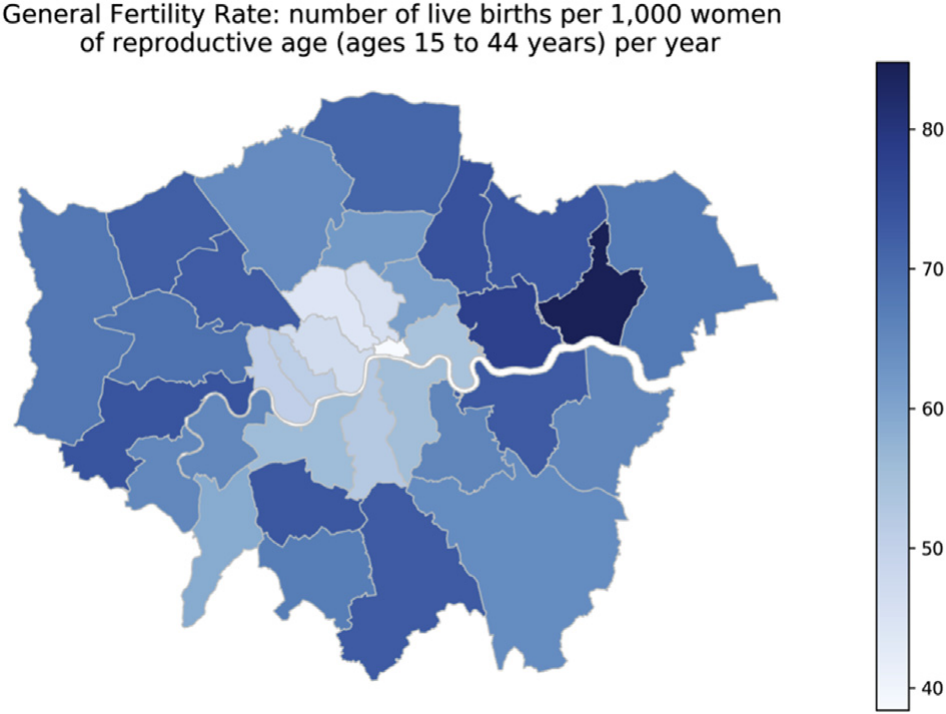
General Fertility Rate mapped at local authority level for Greater London. Source: ONS.

The number of new births simulated in MicroEnv each year is calculated using local authority level General Fertility Rate (GFR) data (the number of live births per 1000 females aged 15–44 per year) [8]. Fig. 7 maps the GFRs by local authority as used within the model.

## 2 Experimental design, materials, and methods

The majority of data compiled in this article for use in MicroEnv are from open source Governmental or Non-Government Organisation (NGO) data repositories. These websites provide annual updates to their data. ONS population data is estimated yearly by applying the ratio of change in consecutive GP Patient Register data to the population found by the previous census [12]. ONS all-cause mortality rates are based on deaths registered in England and Wales. The GBD Results Tool estimates incidence and prevalence rates of IHD using a variety of data sources including surveillance, survey data and claims data – inpatient and outpatient visits [13]. While IHD mortality rates are estimated using vital registration and verbal autopsy data [14]. The GBD employ a Bayesian meta-regression tool (DISMOD-MR 2.1), which combines data from various countries in an integrated manor to provide smooth outputs.

Relative risks for the association between air pollution and rates of IHD incidence, IHD case fatality and all-cause mortality were taken from a recent literature review and meta-analyses [2]. Small area air pollution data for the annual average ambient PM_2.5_ were provided by an environmental consultancy, Ricardo Energy and Environment. This was based on air pollution modelled at 1 × 1 km grid square resolution for the UK using the air dispersion model ADMS and emissions estimates from the UK National Atmospheric Emissions Inventory 2014 [15]. The modelled air pollution was linked to provide an average for each LSOA using GIS methods. Data analysis scripts to produce the graphs and maps in this article were written in Python V3.6 [16] using the geopandas, Basemap and matplotlib libraries. The shapely files used to generate the maps were obtained from the Greater London Authority, who produce GIS files with boundaries at various spatial resolutions [17].
